# Effects of Various Local Antibacterial Preparations on Bacterial Density in Pharyngeal and Tonsillar Mucosa of Patients with Acute Pharyngitis

**DOI:** 10.3390/medicina61122100

**Published:** 2025-11-25

**Authors:** Aigars Reinis, Guna Dansone, Līga Balode, Sandra Gintere, Andrejs Tolstiks, Katrīna Verbovenko, Oļegs Zašibajevs, Taira Safina

**Affiliations:** 1 Department of Biology and Microbiology, Riga Stradins University (RSU), 16 Street Dzirciema, LV-1007 Riga, Latvia; 2 Medical and Clinical Research Department, JSC Olpha, 5 Rupnicu Street, LV-2114 Olaine, Latvia; 3 Ambulatory Clinic, Riga Stradins University (RSU), 16 Street Dzirciema, LV-1007 Riga, Latvia; 4 Faculty of Residency, Riga Stradins University (RSU), 16 Street Dzirciema, LV-1007 Riga, Latvia; 5 Faculty of Medicine, Riga Stradins University (RSU), 16 Street Dzirciema, LV-1007 Riga, Latvia

**Keywords:** acute pharyngitis, throat culture, *Streptococcus pyogenes*, *Staphylococcus aureus*, *Klebsiella* spp., local antibacterial treatment, nitrofurantoin, dequalinium

## Abstract

*Background and Objectives*: Upper respiratory tract infections are one of the most common reasons for outpatient admission. Acute pharyngitis is typically caused by viruses and is self-limiting but in up to 30% of cases, secondary bacterial infection may occur, requiring antibacterial treatment. In the face of growing antibacterial resistance due to inappropriate systemic antibiotic use, different topical therapy would have benefits. The objective of this study was to compare changes in throat and tonsillar mucous membrane bacterial density in patients with acute pharyngitis after a single exposure to a local antibacterial agent presented in different pharmaceutical forms—medicated lozenge, throat spray, or a solution for gargling. *Materials and Methods*: This was a non-interventional observational study that involved 90 adult outpatients with acute pharyngitis. Patients were prescribed to one of three options: medicated lozenges (containing dequalinium chloride and cinchocaine hydrochloride)—Treatment A; throat spray (containing streptocide soluble and norsulfazol-sodium)—Treatment B; or a powder, Furasol^®^ 100 mg (containing furagin soluble), for use as an external gargling solution—Treatment C. Throat swab culture was collected before and 20 min after the single exposure to the medication. Microbial testing was performed using a semi-quantitative quadrant streak plate method to assess bacterial density before and after exposure. *Results*: Four pathogenic agents were isolated from the swabs, with *Staphylococcus aureus* being the most prevalent. Overall, a reduction in post-exposure bacterial growth intensity was observed in 84.6% of the samples after Treatment C exposure, with a statistically significant difference from both Treatment B (57.1%, *p* < 0.05) and Treatment A (10%, *p* < 0.05). The difference was also significant between Treatment A and Treatment B. *Conclusions*: The findings showed that the throat gargling solution had more impact on mucous bacterial load compared to the throat spray and medicated lozenges in the patients with acute pharyngitis. Further research should address the effects of different pharmaceutical forms of the same antibacterial agent, where available.

## 1. Introduction

Upper respiratory tract infections, including acute pharyngitis, are among the most common reasons for outpatient admission, with the highest frequency occurring in children under five years old [1,2]. And they represent a significant burden on healthcare systems and cause substantial absenteeism from work and school [3].

Acute pharyngitis is an inflammation of the mucous membrane of the oropharynx [4]. It is typically caused by viruses such as rhinovirus, adenovirus, respiratory syncytial virus, coronavirus, or influenza. These are self-limiting conditions not requiring any specific therapy. However, secondary bacterial or fungal infection may occur as a complication. More rarely, bacteria may be the pathogen causing a primary respiratory disease. The most important bacterial cause of acute pharyngitis is the beta-hemolytic group A streptococcus (*Streptococcus pyogenes*). These bacteria are found in up to 30% of children and 15% of adults with sore throats. *S. pyogenes* infection should be diagnosed and treated early. If untreated, it may spread out of the throat and induce more severe complications in other organ systems—like the heart, blood vessels, and kidneys. The standard of bacterial pharyngitis diagnostics is a throat culture with 97–100% specificity and 90–95% sensitivity. However, due to the delay in obtaining results, rapid antigen detection test, despite lower sensitivity (70–90%) [5], would be more suitable for routine practice. Also, other clinical scoring criteria like the Centor scale [6] or McIsaac Score [7] may be applied. If bacterial infection by *S. pyogenes* is revealed, systemic antibacterial therapy is recommended [8,9,10,11]. While *S. pyogenes* is the most critical target, other opportunistic bacteria, such as *Staphylococcus aureus* and *Klebsiella pneumoniae*, are also frequently implicated in pharyngeal infections, particularly as secondary infections following a primary viral illness.

Considering growing antimicrobial resistance, topical antibacterial therapy would possess various potential advantages, including high and sustained concentrations of drug directly at the infected site, better compliance, fewer systemic side effects and potentially less of a chance of antimicrobial resistance [12]. Also, this approach could minimize inappropriate use of systemic antibacterials both in children and adults, thus potentially limiting antibiotic-resistant infection spread [13]. Various alternative antiseptic or antibacterial drug forms may be considered to eliminate bacterial pathogens and to prevent medical complications caused by infection.

Locally applied throat sprays, rinses, and medicated lozenges are used for symptomatic bacterial throat infection treatment. Chlorhexidine rinse and spray has been shown to prevent the spread of COVID-19 disease [14]. Also, lozenges containing both antibacterial and anesthetic agents may have benefits in treating acute pharyngitis [15]. The treatments compared in this study represent three distinct approaches: a medicated lozenge (dequalinium chloride), a sulphonamide-based throat spray (streptocide and norsulfazol-sodium), and a nitrofuran-based gargle (furagin soluble). Gargling solution with the active ingredient furagin soluble, which belongs to the nitrofuran group of antibacterials, has been applied in treating sore throat for more than 50 years in the clinical practice in Latvia. It localizes infection and reduces throat pain by affecting bacterial viability and provides pathogenetic treatment in case of acute pharyngitis [16]. Regardless of wide usage of locally applied medicines, there is very limited data available comparing different topical drug forms for sore throat treatment. To address this issue, in our research we compared changes in throat and tonsillar mucous membrane bacterial density in patients with acute pharyngitis after a single exposure to a local antibacterial agent presented in different pharmaceutical forms—a medicated lozenge, throat spray, or solution for gargling.

## 2. Materials and Methods

### 2.1. Subject and Materials

A non-interventional observational study was performed in a general practitioner’s office involving 90 outpatients (61 women and 29 men, 19 to 88 years old). Patients were identified according to specific inclusion criteria. The main criteria were clinically confirmed diagnosis of acute pharyngitis coupled with a prescription for one of three treatments predefined by the study protocol. Exclusion criteria ruled out individuals with an exacerbation of chronic respiratory diseases and/or history of allergies and those who had used any antibiotic during the last 24 h. Before commencing, the study was reviewed and approved by the local Ethics Committee. All participants provided consent for the use of their medical data.

Per protocol treatment options for acute pharyngitis included Decatylen 0.25 mg/0.03 mg lozenges with active ingredients dequalinium chloride, cinchocaine hydrochloride (manufacturer Teva B.V., Haarlem, The Netherlands), Inhalipt spray with active ingredients streptocide soluble, norsulfazol-sodium (manufacturer AS Altajvitamini, Biysk, Russia), or Furasol^®^ 100 mg powder for external solution with active ingredient furagin soluble (manufacturer JSC Olpha, Olaine, Latvia), henceforth referred to as Treatment A, B, and C, respectively.

### 2.2. Methods

The research involved two consecutive microbiological tests. A throat swab culture was collected before and 20 min after a single exposure to the prescribed medication. This 20-min interval was chosen as a practical timeframe to allow for the immediate pharmacodynamic action of all three pharmaceutical forms (including the dissolution time for the lozenge) while minimizing patient burden. Patients were asked to rinse their mouth before the first swab and to avoid eating, drinking, smoking, vaping, or chewing gum between the swabs. Clinical samples were inoculated on Columbia Blood Agar (Liofilchem, Roseto degli Abruzzi, Italy) using a quadrant streak plate technique [17]. This rapid, semi-quantitative scoring method provides a practical assessment of bacterial density and is used as a precedent for this study [18]. After 24–48-h incubation at 37 °C, the grown colonies (Table 1) were identified by VITEK2 analyzer (manufacturer bioMérieux, Craponne, France). To clarify the *in vivo* comparison, the post-exposure growth reduction (PGR) data presented in Table 2 was determined by comparing the semi-quantitative growth score (as defined in Table 1) from the pre-exposure swab to the score from the post-exposure swab for each patient’s identified pathogen. All data were collected using standard laboratory worksheets and handled according to routine laboratory procedure.

#### Statistical Methods and Data Analysis

A formal sample-size calculation was not performed because this study was exploratory and intended to generate hypotheses for future research. The sample size was predetermined to include 30 patients per each group. Pre-exposure presence of bacteria was determined in all clinical samples. The post-exposure growth reduction in pathogenic bacterial species was evaluated by the description shown in Table 1. Growth intensity changes were compared between the treatment groups. Data from all samples with pathogenic bacteria was included in the statistical analysis. Results were presented in absolute and relative frequencies. Where applicable, the two-proportion Z-test was performed at the 5% significance level. This test was used specifically to compare the proportion of samples showing post-exposure growth reduction (PGR) between the three treatment groups (A vs. B, B vs. C, and A vs. C).

## 3. Results

Out of 90 clinical samples, *Staphylococcus aureus* was isolated from 38 (42.2%) samples, *Klebsiella* spp. from 20 (22.2%) samples, *Streptococcus pyogenes* from 10 (11.1%) samples, and *Haemophilus influenzae* from 1 (1.1%) sample. Only normal mucosal microbiota like *viridans* streptococci (*S. mutans*, *S. salivarius*, *S. anginosus*, *S. mitis*, *S. sanguinis*, *S. bovis*), *Neisseria* spp., and *Haemophilus parainfluenzae* were isolated from 22 (22.4%) samples.

In total, 67 of the 90 clinical samples (74.4%) were found to contain one of the four identified pathogens (Table 2). Of these 67 pathogenic samples, a post-exposure growth reduction was observed in 35 cases (53%). In the remaining 31 cases (47%), no change in growth intensity was noted. Importantly, no instance of growth intensification was observed after any treatment.

A post-exposure growth reduction (Table 2) was observed for *S. aureus* and *Klebsiella* spp. in all treatment groups with a significant difference between Treatment B and Treatment C compared to Treatment A. Growth reduction for *S. pyogenes* was observed with Treatment B and Treatment C without a significant difference but not at all with Treatment A. Treatment B did not demonstrate any difference for *H. influenzae* in the single paired sample.

The individual pre- and post-exposure bacterial growth intensity for each pathogenic isolate were assessed using the semi-quantitative quadrant streak method described in Table 1. Bacterial density on the plate media in PGR samples before the treatment exposure ranged from +4 to 1+ for *S. aureus*, from 3+ to 1+ for *Klebsiella* spp., and from +3 to 2+ for *S. pyogenes*. The post-exposure density was 1+, from 1+ to ½+, and from 2+ to 1+, respectively (Table 3). Quantitatively, in most of the cases (72.2%), bacterial density was reduced by one quadrant (↓ 1+); less frequently (22.2%), by two quadrants (↓ 2+); and in a single case each, by 0.5 (↓ ½+) or by three (↓ 3+) quadrants.

**Figure 1:**
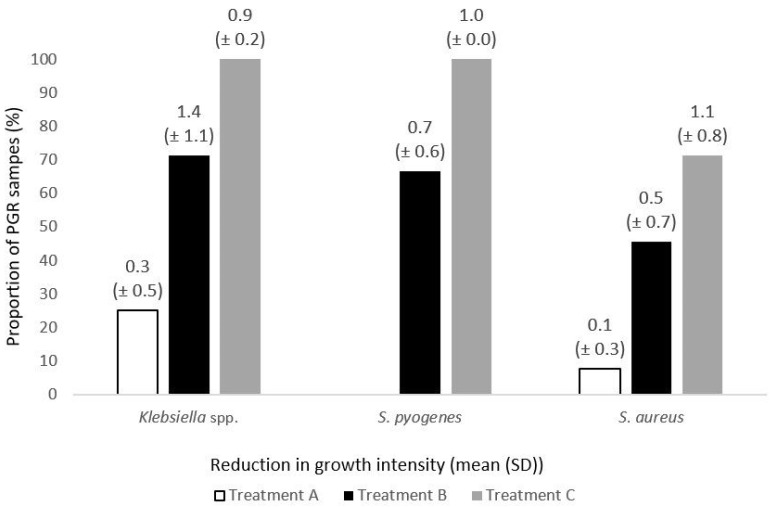
The post-exposure reduction in bacterial density. The mean growth reduction measured in quadrants (Q) was 0.3 (±0.5) after a single exposure to Treatment A (medicated lozenges), 1.4 (±1.1) after Treatment B (throat spray), and 0.9 (±0.2) after Treatment C (gargling solution) for *Klebsiella* species. For *S. aureus* it was 0.1 (±0.3), 0.5 (±0.7), and 1.1 (±0.8), respectively. In case of *S. pyogenes*, a negligible post-exposure growth reduction was observed only for Treatment A (medicated lozenges) and Treatment C (gargling solution)—0.3 (±0.5) Q and 0.5 (±0.3) Q, respectively.

The mean growth reduction was around one quadrant for all treatments, except for *S. pyogenes* with Treatment A (no reduction) and for *S. aureus* with Treatment B (1.2) and Treatment C (1.4) (Figure 1).

## 4. Discussion

Pathogenic bacteria remain important in acute pharyngitis either as a primary causative agent or a secondary infection. *S. pyogenes* is the most common cause of bacterial pharyngitis. Early eradication of this pathogen is important to reduce morbidity and the risks of potentially serious medical complications such as rheumatic fever and acute glomerulonephritis. Other bacteria commonly involved in upper respiratory tract infections are *Staphylococcus aureus*, *Haemophilus influenza*, *Klebsiella pneumoniae*, *Proteus*, *Enterobacter*, and *Citrobacter* species. In addition, it should be considered that the host’s immunity is lowered during viral pharyngitis, which, in turn, may increase the risk of secondary bacterial complications originating even from opportunistic microorganisms [8,19].

In our study, the following species of pathogenic bacteria were isolated—*Staphylococcus aureus*, *Streptococcus pyogenes*, *Haemophilus influenza* (one isolate together with Klebsiella), and *Klebsiella* spp. (mainly *Klebsiella pneumoniae*).

Overall, a reduction in post-exposure bacterial growth intensity was observed in 84.6% of samples after Furasol^®^ exposure. A statistically significant difference was observed between Furasol^®^ compared to Inhalipt (57.1%, *p* < 0.05) and Decatylen (10%, *p* < 0.05). There was also a significant difference between Decatylen and Inhalipt (*p* < 0.05).

*S. aureus* is a major cause of community-acquired and nosocomial infections, bacteraemia, and infective endocarditis, as well as skin, soft tissue, and pleuropulmonary infections [20]. According to data in the literature, 90–100% of *S. aureus* strains are sensitive to nitrofurans [21,22]. In our study, post-exposition bacterial growth intensity reduction was observed in 71.4% of clinical samples in the Furasol^®^ group, in a lesser portion of the Inhalipt group (45.5%), and in few samples in the Decatylen group (7.7%). Although sulphonamides (active substance of Inhalipt) are generally effective against staphylococci, the reduced efficacy compared with Furasol^®^ could be partially explained by the different modes of application. Furasol^®^ in a form of a solution for gargling provides a good “flushing” effect in addition to its antibacterial activity. Meanwhile Inhalipt was applied by spraying, and more microorganisms, especially those in biofilms, remained untargeted.

In general, streptococci are listed among microorganisms susceptible to nitrofurans. However, there are few scientific studies about sensitivity to or resistance against *S. pyogenes*. This is apparently because nitrofurans are mainly used to treat urinary tract infections, where the most common target is *S. agalactiae*. In our study, *S. pyogenes* was reduced in all samples in the Furasol^®^ group (100%) and in 66.7% in the Inhalipt group. Decatylen did not show any effect.

The increased prevalence of *K. pneumoniae* infections and resistance rates is a current cause for concern. The resistance rate to nitrofurantoin varies significantly among the *K. pneumoniae* strains isolated from different biologic sample sources (blood, urine, sputum, and cervix), ranging from 13 to 33% [23,24]. Consistent resistance to sulphonamides was reported globally [25]. Resistance rates in *K. pneumoniae* strains from the pharynx are scarce. In our study, Furasol^®^ demonstrated efficacy in all isolates (100%) compared to 71.4% for Inhalipt and 25% for Decatylen. Again, the type of application could play a role—flushing away a “surface pathogen” instead of targeting bacteria that have infected internal tissues and organs.

The significant differences in the efficacy observed are best explained by the diverging pharmacodynamic properties of the three pharmaceutical forms. The superior action of Treatment C (gargling solution) is strongly linked to its dual-action mechanism: the chemical-antibacterial effect of soluble furagin and the significant mechanical ‘flushing’ effect of the gargling process itself. This mechanical effect likely removes a large volume of non-adherent bacteria, mucus, and debris from the mucosal surface, an advantage not shared by the other applied forms. Conversely, the Treatment A (medicated lozenge) relies on prolonged contact time as it dissolves for its antiseptic (dequalinium) to act. Its very poor performance (10% reduction) observed in our study suggests that this mechanism is less effective in an *in vivo* setting, potentially due to the agent’s limited ability to penetrate pharyngeal biofilms, a noted limitation of dequalinium. Treatment B (throat spray) represents an intermediate form, with the drug applied topically but lacking both the comprehensive mechanical flushing of the gargle and the prolonged contact time of the lozenge.

*H. influenza* was detected only in one sample in the Inhalipt group and did not show signs of reduction after administration of the throat spray.

Dequalinium (in medicated lozenge) is absorbed into the bacterial cell surface and diffused through the cell wall disrupting its permeability. In bacteria, it denatures proteins involved in respiration and glycolysis, interfering with bacterial metabolism and protein synthesis. Dequalinium inhibits mitochondrial ATP synthesis and blocks glucose metabolism [26]. These different modes of action make the development of antimicrobial resistance to the substance unlikely [27]. Thus, topical application of the agent in nasopharyngitis would demonstrate a good antibacterial effect against the bacteria isolated during our study. However, the action seemed to be rather weak. This was probably due to the fact that biofilm-forming microorganisms play an important role in bacterial diseases of the oral cavity and pharynx. But dequalinium has limited or no efficacy toward mature biofilms [28].

All three products used in our study demonstrated antibacterial activity. However, the higher activity of Furasol^®^ could be explained by its mode of application, i.e., mechanical (“flushing”) effect on top of its antibacterial action compared to the throat spray and medicated lozenge. In the literature, only one publication was found that compared different pharmaceutical forms used for the treatment of bacterial pharyngitis with no differences observed [29]. However, there are data confirming the role of throat gargling in limiting upper respiratory tract infections, reducing total duration of illness, systemic medication use, and contact transmission [30,31].

When considering the clinical relevance of the obtained results, it is important to take into account other factors such as time required to reduce the bacterial density, effect on mucosal regeneration, moisturizing, softening effect, and schedule of administration characterizing certain medicinal products, as well as patient preferences. When evaluating the obtained results, the limitations of the study must also be considered. The principle of blinding was not applied; therefore, a risk of bias exists at the microbiological evaluation stage, particularly during the semi-quantitative reading of the growth intensity. Further-more, as this was an observational study, the precise volume and duration of the gargling process for Treatment C were not strictly standardized or recorded, which presents another limitation.

## 5. Conclusions

The results of our study showed that a solution for gargling provided higher reduction in bacterial density in pharyngeal and tonsillar mucosa in patients with acute pharyngitis compared to throat spray or medicated lozenges. Further research should address different pharmaceutical forms of the same antibacterial, where such forms are available.

## Figures and Tables

**Table 1:** Description of bacterial growth intensity [17].

Growth Intensity	Description
(½+) scant growth	1–5 colonies
(1+) small growth	growth only in quadrant 1 *
(2+) moderate growth	growth in quadrants 1 and 2 *
(3+) heavy growth	growth in quadrants 1, 2, and 3 *
(4+) very heavy growth	growth in all 4 quadrants

* Do not account for a few colonies in the next quadrant.

**Table 2:** Summary of post-exposure growth reduction in pathogenic bacteria species.

Species	Treatment A		Treatment B		Treatment C		*p* < 0.05
	Paired Samples *n*	PGR *n* (%)	Paired Samples *n*	PGR *n* (%)	Paired Samples *n*	PGR *n* (%)	
*S. aureus*	13	1 (7.7)	11	5 (45.5)	14	10 (71.4)	A/B, A/C
*Klebsiella* spp.	4	1 (25.0)	7	5 (71.4)	9	9 (100.0)	A/C
*S. pyogenes*	4	0 (0.0)	3	2 (66.7)	3	3 (100.0)	-
*H. influenzae*	0	-	1	0 (0.0)	0	-	-
Total	20	2 (10)	21	12 (57.1)	26	22 (84.6)	A/B/C

(PGR) Samples with post-exposure growth reduction.

**Table 3:** *In vitro* growth intensity of pathogenic bacteria from paired clinical samples.

Treatment A			Treatment B			Treatment C		
Species	(a)	(b)	Species	(a)	(b)	Species	(a)	(b)
*Klebsiella* spp.	2+	2+	*Klebsiella* spp.	4+	1+	*Klebsiella* spp.	3+	2+
*Klebsiella* spp.	2+	2+	*Klebsiella* spp.	3+	1+	*Klebsiella* spp.	3+	2+
*Klebsiella* spp.	2+	2+	*Klebsiella* spp.	3+	1+	*Klebsiella* spp.	3+	2+
*Klebsiella* spp.	2+	1+	*Klebsiella* spp.	1+	1+	*Klebsiella* spp.	2+	1+
*S. pyogenes*	3+	3+	*Klebsiella* spp.	2+	1+	*Klebsiella* spp.	2+	1+
*S. pyogenes*	3+	3+	*Klebsiella* spp.	2+	2+	*Klebsiella* spp.	2+	1+
*S. pyogenes*	3+	3+	*Klebsiella* spp.	3+	1+	*Klebsiella* spp.	2+	1+
*S. pyogenes*	2+	2+	*S. pyogenes*	3+	2+	*Klebsiella* spp.	2+	1+
*S. aureus*	3+	3+	*S. pyogenes*	2+	1+	*Klebsiella* spp.	1+	0.5+
*S. aureus*	3+	3+	*S. pyogenes*	2+	2+	*S. pyogenes*	3+	2+
*S. aureus*	3+	3+	*S. aureus*	3+	2+	*S. pyogenes*	3+	2+
*S. aureus*	3+	3+	*S. aureus*	3+	2+	*S. pyogenes*	2+	1+
*S. aureus*	3+	3+	*S. aureus*	3+	2+	*S. aureus*	4+	3+
*S. aureus*	3+	3+	*S. aureus*	3+	1+	*S. aureus*	3+	2+
*S. aureus*	3+	3+	*S. aureus*	2+	1+	*S. aureus*	3+	2+
*S. aureus*	2+	2+	*S. aureus*	2+	2+	*S. aureus*	3+	1+
*S. aureus*	2+	2+	*S. aureus*	2+	2+	*S. aureus*	3+	1+
*S. aureus*	2+	2+	*S. aureus*	2+	2+	*S. aureus*	3+	1+
*S. aureus*	2+	2+	*S. aureus*	2+	2+	*S. aureus*	3+	1+
*S. aureus*	2+	2+	*S. aureus*	2+	2+	*S. aureus*	2+	2+
*S. aureus*	2+	1+	*S. aureus*	2+	2+	*S. aureus*	2+	2+
			*H. influenzae*	3+	3+	*S. aureus*	2+	2+
						*S. aureus*	2+	1+
						*S. aureus*	2+	1+
(a)—pre-exposure growth						*S. aureus*	2+	1+
(b)—post-exposure growth						*S. aureus*	1+	1+

## Data Availability

The original contributions presented in this study are included in the article. Further inquiries can be directed to the corresponding author.

## References

[1] Finley C.R., Chan D.S., Garrison S., Korownyk C., Kolber M.R., Campbell S., Eurich D.T., Lindblad A.J., Vandermeer B., Allan G.M. (2018). What are the most common conditions in primary care?. Can. Fam. Physician.

[2] Jin X., Ren J., Li R., Gao Y., Zhang H., Li J., Zhang J., Wang X., Wang G. (2021). Global burden of upper respiratory infections in 204 countries and territories, from 1990 to 2019. E Clin. Med..

[3] Pfoh E., Wessels M.R., Goldmann D., Lee G.M. (2008). Burden and economic cost of group A streptococcal pharyngitis. Pediatrics.

[4] Calderaro A., Buttrini M., Farina B., Montecchini S., De Conto F., Chezzi C. (2022). Respiratory tract infections and laboratory diagnostic methods: A review with a focus on syndromic panel-based assays. Microorganisms.

[5] Shulman S.T., Bisno A.L., Clegg H.W., Gerber M.A., Kaplan E.L., Lee G., Martin J.M., Van Beneden C. (2012). Clinical practice guideline for the diagnosis and management of group A streptococcal pharyngitis: 2012 update by the Infectious diseases society of America. Clin. Infect. Diseases.

[6] NICE Guidance (2019). Rapid Tests for Group a Streptococcal Infections in People with a Sore Throat.

[7] Reinholdt K.B., Rusan M., Hansen P.R., Klug T.E. (2019). Management of sore throat in Danish general practices. BMC Fam. Pract..

[8] Hendaus M.A., Jomha F.A., Alhammadi A.H. (2015). Virus-induced secondary bacterial infection: A concise review. Ther. Clin. Risk Manag..

[9] Alcaide M.L., Bisko A.L. (2007). Pharyngitis and epiglottitis. Infect. Dis. Clin. N. Am..

[10] Sykes E.A., Wu V., Beyea M.M., Simpson M.T.W., Beyea J.A. (2020). Pharyngitis: Approach to diagnosis and treatment. Can. Fam. Physician.

[11] Pichichero M.E. (2025). Treatment and Prevention of Streptococcal Pharyngitis in Adults and Children.

[12] Ray P., Singh S., Gupta S. (2019). Topical antimicrobial therapy: Current status and challenges. Indian J. Med. Microbiol..

[13] Fleming-Dutra K.E., Hersh A.L., Shapiro D.J., Bartoces M., Enns E.A., File T.M., Finkelstein J.A., Gerber J.S., Hyun D.Y., Linder J.A. (2016). Prevalence of inappropriate antibiotic prescriptions among US ambulatory care visits, 2010–2011. JAMA.

[14] Huang Y.H., Huang J.T. (2021). Use of chlorhexidine to eradicate oropharyngeal SARS-CoV-2 in COVID-19 patients. J. Med. Virol..

[15] Palm J., Fuchs K., Stammer H., Schumacher-Stimpfl A., Milde J., DoriPha Investigators (2018). Efficacy and safety of a triple active sore throat lozenge in the treatment of patients with acute pharyngitis: Results of a multi-centre, randomized, placebo-controlled, double-blind, parallel-group trial (DoriPha). Int. J. Clin. Pract..

[16] Wolford R.W., Goyal A., Belgam Syed S.Y., Schaefer T.J. (2025). Pharyngitis. [Updated 2023 May 1]. StatPearls [Internet].

[17] Leber A.L. (2016). Clinical Microbiology Procedures Handbook.

[18] Cox N.A., Richardson L.J., Cosby D.E., Berrang M.E., Wilson J.L., Harrison M.A. (2017). A four-quadrant sequential streak technique to evaluate *Campylobacter* selective broths for suppressing background flora in broiler carcass rinses. J. Food Saf..

[19] Manohar P., Loh B., Athira S., Nachimuthu R., Hua X., Welburn S.C., Leptihn S. (2020). Secondary bacterial infections during pulmonary viral disease: Phage therapeutics as alternatives to antibiotics?. Front. Microbiol..

[20] González-García S., Hamdan-Partida A., Bustos-Hamdan A., Bustos-Martínez J. (2021). Factors of nasopharynx that favor the colonization and persistence of *Staphyloccocus aureus*. Pharynx—Diagnosis and Treatment.

[21] An N., Hai L.H.L., Luong V.H., Vinh N.T.H., Hoa P.Q., Hung L., Son N.T., Hong L.T., Hung D.V., Kien H.T. (2024). Antimicrobial resistance patterns of *Staphylococcus aureus* isolated at a General Hospital in Vietnam between 2014 and 2021. Infect. Drug Resist..

[22] Gitau W., Masika M., Musyoki M., Museve B., Mutwiri T. (2018). Antimicrobial susceptibility pattern of *Staphyloccocus aureus* isolates from clinical specimens at Kenyatta National Hospital. BMC Res. Notes.

[23] Li G., Zhao S., Wang S., Sun Y., Zhou Y., Pan X. (2019). A 7-year surveillance of the drug resistance in Klebsiella pneumoniae from primary health care center. Ann. Clin. Microbiol. Antimicrob..

[24] Park K.-S., Kim D.R., Baek J.Y., Shin A., Kim K.-R., Park H., Son S., Cho H., Kim Y.-J. (2023). Susceptibility to fosfomycin and nitrofurantoin of ESBL-positive Escherichia coli and Klebsiella pneumoniae isolated from urine of pediatric patients. J. Korean Med. Sci..

[25] Asri N.A.M., Ahmad S., Mohamud R., Hanafi N.M., Zaidi N.F.M., Irekeola A.A., Shueb R.H., Yee L.C., Noor N.M., Mustafa F.H. (2021). Global prevalence of nosocomial multidrug-resistant Klebsiella pneumoniae: A systematic review and meta-analysis. Antibiotics.

[26] Dequalinium. https://go.drugbank.com/drugs/DB04209.

[27] Raba G., Ďurkech A., Malík T., Bassfeld D., Grob P., Hurtado-Chong A., Botta S., Sach A., Golańska-Wróblewska M., Paškala M. (2024). Efficacy of dequalinium chloride vs. metronidazole for the treatment of bacterial vaginosis: A randomized clinical trial. JAMA Netw. Open.

[28] Schwarz S.R., Hirsch S., Hiergeist A., Kirschneck C., Muehler D., Hiller K.-A., Maisch T., Al-Ahmad A., Gessner A., Buchalla W. (2021). Limited antimicrobial efficacy of oral care antiseptics in microcosm biofilms and phenotypic adaptation of bacteria upon repeated exposure. Clin. Oral. Investig..

[29] Radkova E., Burova N., Bychkova V., DeVito R. (2017). Efficacy of flurbiprofen 8.75 mg delivered as a spray or lozenge in patients with sore throat due to upper respiratory tract infection: A randomized, non-inferiority trial in the Russian Federation. J. Pain. Res..

[30] Satomura K., Kitamura T., Kawamura T., Shimbo T., Watanabe M., Kamei M., Takano Y., Tamakoshi A. (2005). Prevention of upper respiratory tract infections by gargling: A randomized trial. Am. J. Prev. Med..

[31] Ramalingam S., Graham C., Dove J., Morrice L., Sheikh A. (2019). A pilot, open labelled, randomized controlled trial of hypertonic saline nasal irrigation and gargling for the common cold. Sci. Rep..

